# Diabetes Mellitus and Lipoprotein(a): A Determinant Interaction in Micro- and Macrovascular Damage

**DOI:** 10.3390/ijms262311427

**Published:** 2025-11-26

**Authors:** Alberto Polo-Barranco, Carlos Rebolledo-Maldonado, Valeria Esquiaqui-Rangel, Andrea Nuñez-Mejia, Jeisón Rambal-Torres, Valentina Barraza-Ahumada, Shivleivy Vargas-Cantillo, Wylman Benavides-De la Cruz, Valentina Liñán-Martínez, Valentina Rada-Obeso, Muna Isaac-Escorcia, Neil Martínez-Fontalvo, José Correa-Guerrero, Dairo Rodelo-Barrios, Elber Osorio-Rodríguez

**Affiliations:** 1 Department of Internal Medicine, Clínica Iberoamérica, Barranquilla 080002, Colombia; dralbertopolob@gmail.com (A.P.-B.); dra.munaisaace@gmail.com (M.I.-E.); 2 Department of Internal Medicine, Faculty of Medicine, Metropolitana University, Barranquilla 080002, Colombia; jesonramball@hotmail.com (J.R.-T.); valentinalinan12@gmail.com (V.L.-M.); valentinarada2003@gmail.com (V.R.-O.); 3 Critical and Internal Medicine Research Group (CRITIMED), Cartagena de Indias 130005, Colombia; valentinaahu@gmail.com (V.B.-A.); wiilmanbenavides@gmail.com (W.B.-D.l.C.); neilmartinezf18@gmail.com (N.M.-F.); josegabriel2101@gmail.com (J.C.-G.); dairorodelo1992@gmail.com (D.R.-B.); 4 Department of Intensive Medicine, Clínica Iberoamérica, Barranquilla 080002, Colombia; carlos.rebolledo@unisimon.edu.co (C.R.-M.); shivleivyvargas@gmail.com (S.V.-C.); 5 Department of Critical Medicine and Intensive Care, Faculty of Medicine, Simón Bolívar University, Barranquilla 080002, Colombia; valeria.esquiaqui@unisimon.edu.co (V.E.-R.); ancanume0210@gmail.com (A.N.-M.); 6 Group Care Medicine, Clínica Colsanitas, Bogotá D.C. 110931, Colombia; 7 Department of Internal Medicine, University of Cartagena, Cartagena de Indias 130005, Colombia

**Keywords:** diabetes mellitus, lipid-lowering therapy, lipoprotein(a), macrovascular complications, microvascular complications, residual cardiovascular risk

## Abstract

Even when people with diabetes mellitus (DM) meet their cholesterol goals, they still face a higher risk of heart and blood vessel problems. One major reason is a particle called lipoprotein(a), or Lp(a), which is similar to LDL cholesterol. Raised levels of Lp(a) are inherited rather than caused by lifestyle. Lp(a) can build up in the body and make it easier for blood clots to form because it closely resembles a protein called plasminogen, reducing its ability to form plasmin that dissolves blood clots. At the same time, chemical changes like oxidation and glycation can make blood vessels more inflamed, adding to the risk. Elevated concentrations of Lp(a) (>30 mg/dL; 75 nmol/L), and particularly >50 mg/dL (125 nmol/L), are independently associated with coronary artery disease, ischemic stroke, diabetic nephropathy, retinopathy, and neuropathy. Conventional lipid-lowering therapies exert neutral or modest effects on Lp(a), in contrast to RNA-based targeted agents (antisense oligonucleotides and siRNA [Small Interfering RNA]), which achieve reductions of 70–95% and show consistent results in Phase 2 clinical trials. In this review, we bring together findings from laboratory research and clinical studies, and highlight why it is important to measure Lp(a) levels—at least once in a person’s life, and especially in those with diabetes—to help doctors better assess risk and plan more effective treatments. In diabetic populations, the adaptation of Lp(a)-targeted therapies could redefine the management of residual risk and improve both cardiovascular and microvascular outcomes.

## 1. Introduction

Dyslipidemia is a well-known risk factor for atherosclerotic cardiovascular disease (ASCVD) in people with diabetes mellitus (DM) [1,2]. About 800 million people worldwide have diabetes, which greatly raises the risk of major adverse cardiovascular events (MACE) [3]. Individuals with DM are two to four times more likely to develop ASCVD than those without diabetes [4,5]. About 35–50% of subjects with type 2 diabetes (T2DM) have dyslipidemia, making early diagnosis and timely treatment important [6,7]. Even when lipid targets are met, some risk remains, so improved risk assessment is needed to further reduce MACE [8,9].

Dyslipidemia is a disorder of lipoprotein metabolism, caused by either too much production or poor removal of lipoproteins [10]. In DM, the most common type of atherogenic dyslipidemia involves lower high-density lipoprotein cholesterol (HDL-C), higher triglycerides, and more small dense low-density lipoprotein (sdLDL) particles [1,11]. Glycemic control alone does not prevent ASCVD, since insulin resistance leads to more production of triglyceride-rich very-low-density lipoprotein (VLDL) particles, greater cholesteryl ester transfer protein (CETP) activity, and higher hepatic lipase activity [12,13,14]. As a result, early diagnosis and prevention of MACE depend on measuring these harmful lipid levels [15].

Lipoprotein(a) [Lp(a)] is an LDL-like particle that is especially important in DM because it is strongly linked to atherogenesis and cardiovascular events [16]. Its structure contains apolipoprotein(a) [apo(a)], which is 75–94% similar to plasminogen and gives Lp(a) its proatherothrombotic properties [17,18,19,20]. In DM, these features are linked to a higher risk of coronary heart disease and ischemic stroke [21,22,23,24]. High levels of Lp(a), especially above 50 mg/dL (125 nmol/L), are associated with faster atherosclerosis and greater ASCVD risk [20].

Recent studies show that LDL and other lipoproteins, including Lp(a), can be changed by glycation and oxidation, which speeds up atherogenesis and diabetic vasculopathy [25,26,27]. Long-term high blood sugar causes non-enzymatic glycation of proteins and lipids, leading to advanced glycation end-products (AGEs) [28]. For lipoproteins, this process affects apolipoprotein B [apoB] and surface phospholipids, both key parts of Lp(a) [29]. This harmful process is made worse by oxidative stress, chronic inflammation, cell death, and the growth of vascular smooth muscle cells [30,31,32].

Despite progress, there are still major gaps in our understanding of Lp(a) biology in DM [33]. In this review, we bring together molecular and clinical insights on Lp(a) in DM and discuss its role in both microvascular and macrovascular complications.

## 2. Chemical Structure of Lipoprotein(a)

**Figure 1:**
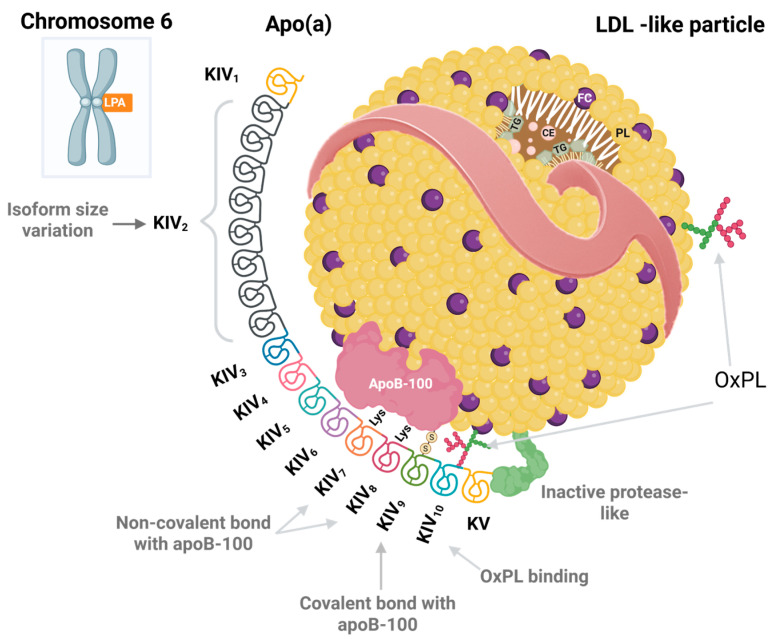
Schematic representation of the LPA gene locus on chromosome 6 (band 6q27) and the structure of lipoprotein(a) [Lp(a)]. Lp(a) consists of an LDL particle containing apolipoprotein B100 (apoB100) covalently linked via a disulfide bond (–S–S–) to apolipoprotein(a) [apo(a)]. Apo(a) is composed of multiple kringle domains—kringle IV (subtypes 1–10) and kringle V—and a catalytically inactive protease domain. The number of kringle IV type 2 (KIV-2) repeats varies widely among individuals and determines apo(a) size and, consequently, plasma Lp(a) concentrations. Abbreviations: LPA: apolipoprotein(a) gene; apoB100: apolipoprotein B100; apo(a): apolipoprotein(a); KIV: kringle IV; KIV-1: kringle IV type 1; KIV-2: kringle IV type 2; KIV-3: kringle IV type 3; KIV-4: kringle IV type 4; KIV-5: kringle IV type 5; KIV-6: kringle IV type 6; KIV-7: kringle IV type 7; KIV-8: kringle IV type 8; KIV-9: kringle IV type 9; KIV-10: kringle IV type 10; KV: kringle V; OxPL: Oxidized phospholipids. Created with https://BioRender.com/wps73bu (accessed on 31 October 2025).

Lp(a) is a complex plasma lipoprotein particle composed of two major components covalently linked to each other [34]. The first is an LDL-like particle, with a cholesterol ester- and triglyceride-rich core surrounded by a phospholipid monolayer, whose structural protein is apolipoprotein B100 (apoB100) [35]. The second is apolipoprotein(a) [apo(a)], a large glycoprotein covalently bound to apoB100 via a disulfide bond [36]. Apo(a), which shares high sequence homology with plasminogen, is composed of multiple kringle-type domains—Kringle IV (KIV) [subtypes (1–10)] and Kringle V(KV)—along with a catalytically inactive protease-like domain [37]. This unique architecture underlies the distinctive biochemical properties of Lp(a), as illustrated in Figure 1.

Apo(a) exhibits marked size variability determined by the number of Kringle IV type 2 (KIV-2) repeats [38,39]. The isoform size of apo(a) is the principal determinant of circulating Lp(a) concentrations [37]. Large isoforms, which contain more KIV-2 repeats, fold and mature more slowly within the endoplasmic reticulum (ER); consequently, they are retained for longer periods, and a proportion undergoes degradation before secretion [40]. This reduces the effective production rate of Lp(a) and translates into lower plasma levels [41]. In contrast, small isoforms are more efficiently synthesized and secreted, resulting in higher circulating concentrations [42]. This variability is primarily explained by genetic determinants at the LPA locus, located on the long arm of chromosome 6 (6q27) [43]. Moreover, multiple single-nucleotide polymorphisms (SNPs) within the LPA locus modulate plasma Lp(a) levels, while other genes, such as APOE and APOH, contribute to interindividual variability to a lesser extent [43,44].

Sex-related differences have also been described: women generally exhibit slightly higher Lp(a) levels compared with men (~5–10%) [39,45]. This difference becomes more pronounced after menopause (up to ~17%), likely mediated by hormonal factors [46,47]. Nonetheless, the genetic determinants of Lp(a)—including codominant inheritance of LPA alleles, SNPs, and the number of KIV-2 repeats—operate similarly in both sexes, although their phenotypic expression may be modulated by age and hormonal environment [48].

## 3. Metabolism of Lipoprotein(a)

### 3.1. Biosynthesis

**Figure 2:**
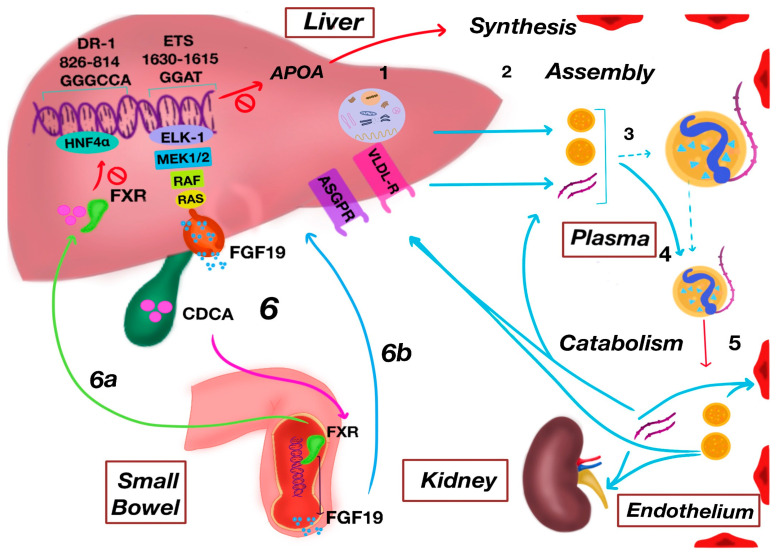
Schematic model of Lp(a) metabolism: (1) hepatic synthesis of apo(a); (2) assembly of apo(a) with lipoprotein particles at the hepatocyte surface/space of Disse or in plasma; (3) lipid association on triglyceride-rich (VLDL-like) or cholesterol-rich (LDL-like) cores; (4) intravascular remodeling converting VLDL-like Lp(a) toward an LDL-like phenotype; (5) catabolism: partial dissociation of Lp(a) and apo(a) proteolysis by elastases and matrix metalloproteinases within the vascular wall, followed by hepatic clearance (ASGPR, LDLR), renal uptake, and vascular deposition; (6) FXR-mediated transcriptional repression of LPA via two complementary routes: (6a) the intestinal–hepatic axis (ileal FXR → FGF19 → FGFR4 → RAS–RAF–MEK1/2–ELK-1; modulation of the ETS site −1630/−1615) and (6b) a direct hepatic route (FXR/HNF4α competition at DR-1 −826/−814). Solid arrows indicate chronological biochemical steps, and dashed arrows indicate modulatory or inhibitory interactions. Abreviations: Lp(a): lipoprotein(a); apo(a): apolipoprotein(a); TG: triglyceride; VLDL: very-low-density lipoprotein; LDL: low-density lipoprotein; LDLR: low-density lipoprotein receptor; ASGPR: asialoglycoprotein receptor; FXR: farnesoid X receptor; CDCA: chenodeoxycholic acid; HNF4α: hepatocyte nuclear factor 4 alpha; FGF19: fibroblast growth factor 19; RAS: RAS protein family; RAF: RAF proto-oncogene serine/threonine–protein kinase; MEK1/2: mitogen-activated protein kinase kinase 1/2 (MAPKK1/2); ELK-1: ETS domain-containing protein ELK-1; ETS: E26 transformation-specific transcription factor family. Created by authors.

Apo(a) is synthesized mainly in the liver; the site of Lp(a) assembly has been proposed to be within hepatocytes, in the space of Disse, or even in the plasma compartment [33]. Following translation, the precursor protein undergoes maturation in the ER, where it experiences N-glycosylation, folding, and proteasomal degradation [37]. In the Golgi apparatus, apo(a) acquires additional carbohydrate chains through both N- and O-glycosylation, ultimately reaching a molecular mass of 300–800 kDa [37,49]. Since plasma Lp(a) concentrations are largely determined by the rate of apo(a) synthesis, transcriptional regulation of the LPA gene plays a central role in its control [50]. In hepatocytes, transcription is influenced by hepatocyte nuclear factor 4 alpha (HNF4α) and modulated by nuclear receptors, particularly the farnesoid X receptor (FXR), which responds to bile acids such as chenodeoxycholic acid (CDCA) [51]. FXR activation represses apo(a) expression through two mechanisms: (i) directly displacing HNF4α from the Downregulator of Transcription 1 (DR-1) element (–826/–814) within the LPA promoter and (ii) indirectly inducing intestinal fibroblast growth factor 19 (FGF19), which activates hepatic FGFR4 signaling. This triggers the RAS–RAF–MEK1/2 cascade, leading to ELK-1 phosphorylation and binding to an ETS site (–1630/–1615) in the LPA promoter, thereby suppressing apo(a) transcription [50,52,53]; see Figure 2.

### 3.2. Assembly

Under physiological conditions, more than 95% of circulating apo(a) is covalently bound to LDL particles, which predominantly arise from the intravascular lipolysis of VLDL [54]. In vitro, Lp(a) can be reconstituted by mixing recombinant apo(a) with LDL in the absence of enzymatic cofactors, supporting the extracellular assembly model [50]. In this process, an initial non-covalent interaction occurs: the apo(a) region spanning Kringle IV-7 to IV-8 binds to a charged site at the C-terminal region of apoB100 [37]. Subsequently, a covalent disulfide bond forms between cysteine 4328 in apo(a) (KIV-9) and cysteine 4057 in apoB100 [55,56]. Alternatively, intracellular assembly has been documented in HepG2 cells expressing a minigene of apo(a) [57]. Thus, both extracellular and intracellular mechanisms are considered plausible [37].

### 3.3. Catabolism

Most Lp(a) breakdown happens in the liver [34]. Possible receptors involved are the asialoglycoprotein receptor (ASGP-R) and plasminogen receptors [58]. Studies of familial hypercholesterolemia have demonstrated that, in contrast to LDL, Lp(a) is not primarily removed by the low-density lipoprotein receptor (LDLR) [56]. Other receptors, such as the very-low-density lipoproteins receptor (VLDL-R), can bind Lp(a) in vitro, although their physiological relevance in humans remains uncertain [59]. Animal studies show that 50–60% of labeled Lp(a) ends up in the liver, with the rest found in bile, kidneys, and spleen [60].

In the kidneys, there is about a 10% difference in Lp(a) concentration between arteries and veins [61]. Healthy people have been found to excrete apo(a) fragments in their urine, ranging from 50 to 160 kDa [62]. While it is not clear exactly where these fragments are made, evidence suggests many come from outside the kidneys and are then filtered out by them [50]. Urinary apo(a) fragments immediately decrease when plasma Lp(a) is reduced by LDL apheresis, indicating a close relationship between plasma and renal handling of Lp(a) [63].

## 4. Behavior of Lipoprotein(a) According to Diabetic Phenotype

Diabetic dyslipidemia is a complex set of quantitative and qualitative lipid and lipoprotein abnormalities with strong atherogenic potential [64]. Plasma levels of Lp(a) are primarily determined by genetic factors, although environmental and clinical conditions can modify them [65]. Sex steroids and hormone replacement therapy can decrease Lp(a) [66,67,68], whereas hypothyroidism is usually associated with elevated levels [69]. In healthy subjects, an intravenous glucose tolerance test (IVGTT) revealed an inverse relationship between Lp(a) and first-phase insulin secretion [70], suggesting a possible interplay between Lp(a) metabolism and pancreatic insulin secretion [71].

Lp(a) levels also vary across different diabetes subtypes [72]. In type 1 diabetes mellitus (T1DM), concentrations are usually comparable to healthy individuals, depending on glycemic control and renal involvement [73]. In T2DM, a paradox has been described in which lower Lp(a) levels are observed, attributed to hyperinsulinemia and hormonal alterations [74,75,76]. In maturity-onset diabetes of the young (MODY), Lp(a) levels are influenced by genetic variation in LPA and hepatopancreatic factors [56]. The following subsections describe each phenotype and its influence on plasma Lp(a) concentrations.

### 4.1. Type 1 Diabetes Mellitus

T1DM results from the interaction of genetic, environmental, and immunological factors, culminating in autoimmune destruction of pancreatic β-cells and insulin deficiency [77,78]. In T1DM, glycemic control is closely associated with Lp(a): higher values correlate with elevated glycosylated hemoglobin (HbA1c) [79]. In a Japanese pediatric cohort, increased Lp(a) strongly correlated with HbA1c [80]. A plausible mechanism is that non-enzymatic glycation prolongs lipoprotein half-life, thereby increasing Lp(a) [81]. In addition, elevated levels of Lp(a) have been reported in the presence of micro/macroalbuminuria, contributing to cardiovascular events (ischemic heart disease) [82].

### 4.2. Type 2 Diabetes Mellitus

T2DM combines genetic and environmental determinants and is characterized by hyperglycemia, insulin resistance, and relative impairment of insulin secretion [77]. In this context, Lp(a) metabolism appears to be modulated by insulin [74]. Several studies have reported lower plasma Lp(a) concentrations in T2DM compared with non-diabetic controls [83]. A Brazilian cohort demonstrated an inverse relationship between basal insulin and Lp(a): higher Lp(a) when insulin <10 μIU/mL, and lower Lp(a) when ≥10 μIU/mL [81]. This observation has led to the concept of the “Lp(a) paradox in T2DM” [56]. One proposed mechanism is that chronic hyperinsulinemia negatively regulates Lp(a), either by suppressing its synthesis or enhancing its clearance [75]. However, when Lp(a) levels are elevated, their associated risk is not fully neutralized by the T2DM metabolic context.

### 4.3. Maturity-Onset Diabetes of the Young (MODY)

MODY is characterized by early onset, autosomal dominant inheritance, absence of autoimmunity, and minimal insulin resistance [77]. An inverse relationship exists between Lp(a) and apo(a) isoform size: a higher number of KIV-2 repeats is associated with lower Lp(a) [84]. Lp(a) concentrations are primarily determined by production rate, modulated by the LPA locus [56]. Reduced Lp(a) levels have been described in association with hepatopancreatic gene mutations [85]. For instance, the Q268X variant is linked to decreased apoA-II, apoC-III, and Lp(a), suggesting transcriptional hepatic effects on lipoprotein genes [86]. Mechanistically, the hepatic promoter of LPA contains a DR-1 element recognized by HNF4α; alterations in transcription factors associated with MODY may reduce LPA transcription and Lp(a) synthesis [51,56,87].

## 5. Molecular Mechanisms of Lipoprotein(a) in DM: From Intimal Retention to Lipid Signaling

Poorly controlled diabetes mellitus, characterized by persistent hyperglycemia and insulin resistance, creates an inflammatory vascular microenvironment that contributes to both microvascular and macrovascular complications [88]. Atherogenic dyslipidemia [1,11] induces oxidative stress, endothelial activation, and tissue injury, ultimately resulting in diabetic vasculopathy [89]. Within this context, Lp(a) has emerged as an independent mediator of cardiovascular risk [90,91,92]. These pathological processes involve subendothelial retention, inflammation, and thromboinflammation, which are further intensified by post-translational modifications such as oxidation and glycation, as well as by lipid signaling [93].

### 5.1. Vascular Retention of Lipoprotein(a): Intima, Proteoglycans, and Macrophage Uptake

**Figure 3:**
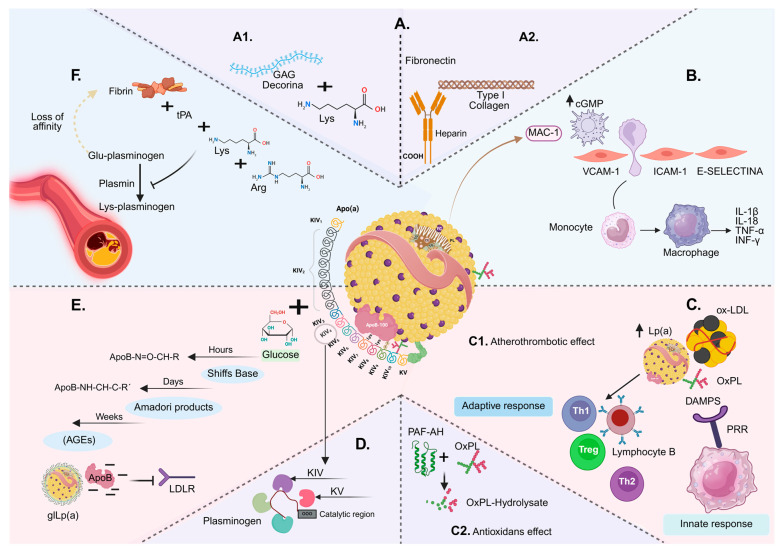
Sequential model of Lp(a) pathophysiology in diabetes mellitus: (**A**) Endothelial and extracellular matrix anchoring: (**A1**) Apo(a) binds decorin/GAGs through lysine sites on apo(a). (**A2**) Subendothelial anchoring via Lp(a)–fibronectin/type I collagen interactions (heparin-facilitated), with possible involvement of MAC-1. (**B**) Endothelial activation and chemotaxis. Upregulation of VCAM-1, ICAM-1, and E-selectin, recruitment of monocytes, and differentiation into macrophages releasing IL-1β, IL-18, TNF-α, and IFN-γ. (**C**) Lp(a)-bound oxidized phospholipids (oxPL). Elevated Lp(a) and its oxPL act as DAMPs that trigger PRR, promoting innate responses and modulating the adaptive arm: (**C1**) Atherothrombotic effect. (**C2**) Antioxidant effect: PAF-AH on Lp(a) hydrolyzes oxPL (oxPL-hydrolysate). (**D**) Structural homology between Lp(a) and plasminogen. KIV/KV domains in apo(a) mimic plasminogen and compete for lysine-binding sites, impairing fibrinolysis (an antifibrinolytic action). (**E**) Non-enzymatic glycation of Lp(a). Glucose forms Schiff bases (hours) and Amadori products (days), evolving to AGEs (weeks); glycation of apoB-100 reduces LDLR binding, favoring gLp(a). (**F**) Additional atherothrombotic/antifibrinolytic potential. Lp(a)/apo(a) occupancy of lysine sites diminishes plasminogen binding and tPA activation, reducing fibrin affinity and promoting thrombo-resistance. Solid arrows indicate chronological biochemical steps, dashed arrows indicate modulatory or inhibitory interactions, and the upward arrow (↑) denotes a relative increase (e.g., in concentration, expression, or activity of the indicated component or pathway). Abbreviations: Lp(a): lipoprotein(a); apo(a): apolipoprotein(a); GAG: glycosaminoglycan; MAC-1: αMβ2 integrin; VCAM-1: vascular cell adhesion molecule-1; ICAM-1: intercellular adhesion molecule-1; E-selectin: endothelial-selectin; IL-1β. interleukin-1 beta; IL-18: interleukin-18; TNF-α: tumor necrosis factor-alpha; IFN-γ: interferon-gamma; oxPL: oxidized phospholipid; DAMPs: damage-associated molecular patterns; PRRs: pattern recognition receptors; PAF-AH: lipoprotein-associated phospholipase A2 (Lp-PLA2); KIV/KV: apo(a) kringle IV/kringle V; AGEs: advanced glycation end products; LDLR: low-density lipoprotein receptor; tPA: tissue plasminogen activator; gLp(a): glycated Lp(a). Created with https://BioRender.com/uenhksj (accessed on 31 October 2025).

Retention is an important early step in starting vascular inflammation on the arterial intima [94]. This happens through lysine binding sites on apo(a) [95] and heparin-binding domains [96]. There are two main pathways: first, apoB-100 binds to glycosaminoglycan chains (decorin); second, apo(a) binds to the proteoglycan core protein [97]. Lp(a) can form complexes with α-defensins and stay outside cells [98], or it can be taken up by macrophages and help form foam cells [99,100]. When Lp(a) is retained under the endothelium with glycosaminoglycans and fibronectin, it stays longer and helps start inflammation and proatherogenic processes in diabetes [101]; see Figure 3.

### 5.2. Early Inflammatory Activation: Endothelium, Adhesion, and Chemotaxis

When Lp(a) is retained, it triggers endothelial signaling pathways and raises the levels of vascular cell adhesion molecule-1 (VCAM-1), E-selectin, and intercellular adhesion molecule-1 (ICAM-1) [102], after activating the transcription factor nuclear factor kappa B (NF-κB) [103]. These inflammatory molecules interact with integrin Mac-1, which helps guide chemotaxis and the transformation of monocytes into macrophages through the cyclic guanosine monophosphate pathway [103,104]. Once activated, these monocytes become macrophages and increase the production of interleukin-1β (IL-1β), interleukin-18 (IL-18), tumor necrosis factor-α (TNF-α), and interferon-γ (IFN-γ) [105,106]. In the presence of type I collagen and apo(a), they favor reactive oxygen species (ROS) and matrix metalloproteinase 9 (MMP-9), which configures a prothrombotic, proinflammatory, and procalcifying microenvironment that accelerates layer instability and promotes rupture and vascular damage [107,108].

### 5.3. Lipoprotein(a)–Oxidized Phospholipid Axis: Signaling and Immune Amplification

Once LDLs migrate to the vascular intima, they undergo a transformation that generates oxidized LDL (oxLDL) and oxidized phospholipids (OxPLs) [109,110,111]. Lp(a) binds and transports proinflammatory OxPLs, which are essential in atherogenesis [112]. These OxPLs act as damage-associated molecular patterns (DAMPs) that activate pattern recognition receptors (PRRs) [92], amplifying the innate immune response. Furthermore, they promote the polarization of adaptive responses mediated by helper T cells (Th1, Th2, and Th17), regulatory T cells (Treg), and B cells [113]. In addition, OxPLs interact with scavenger receptors (CD36, SR-B1), Toll-like receptors (TLR2/4/7), G protein-coupled receptors (GPCR, EP2), and peroxisome proliferator-activated receptors (PAF-R, PPARγ, and PPARγ/α), increasing signaling for the stimulation of cytokines, chemokines, and adhesion molecules, which leads to greater plaque instability [114].

This dual role is paradoxical: at low levels, Lp(a) can exert an antioxidant role through the enzyme platelet-activating factor acetylhydrolase (PAF-AH), capable of hydrolyzing and inactivating platelet-activating factor (PAF) and OxPLs [115]; at high levels, its activity decreases, favoring accumulation of OxPLs and ASCVD [116,117,118]. This effect is enhanced in DM by oxidative stress and glycation, which increase the OxPL load and decrease detoxifying capacity, closing a proatherogenic vicious circle [119,120,121]. The quantification of OxPL-apoB and OxPL-apo(a) has been proposed as a marker of vascular inflammation and pharmacodynamic endpoint in interventions that reduce Lp(a), providing a mechanistic window between OxPL burden and atherogenic activity [122,123,124]. See Figure 3.

### 5.4. Non-Enzymatic Protein Glycation

Persistent hyperglycemia promotes the production of AGEs [125]. In Lp(a), reducing sugars bind to the amino-terminal groups of proteins such as apoB and nucleic acids, forming unstable Schiff bases that are then converted into Amadori products and finally into AGEs [126]. Glycation changes the properties of Lp(a) by modifying its electrical charge, as it alters the lysine and arginine in apoB, which reduces its electropositivity, decreases its affinity for LDLR, and affects its clearance [127,128]. The LDL/Lp(a) glycated complex accumulates in the arterial wall and is taken up by receptor for advanced glycation end-products (RAGEs), TLR, and scavenger receptors, promoting foam cell formation during DM [127,129].

**Figure 4:**
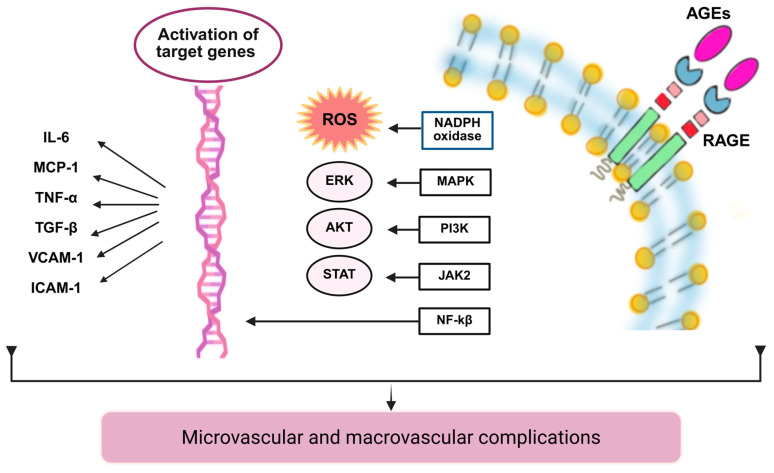
Non-enzymatic glycation of Lp(a) and proinflammatory signaling. Lp(a) undergoes non-enzymatic glycation (Maillard reaction) with sequential formation of Schiff bases → Amadori products → AGEs. Glycated Lp(a) becomes more electronegative and loses affinity for LDLR. AGE–RAGE engagement activates inflammatory pathways—NADPH oxidase/ROS, JAK2/STAT, PI3K/AKT, MAPK/ERK, and NF-κB— driving transcriptional activation of cytokines (IL-6, TNF-α), chemokines (MCP-1), growth factors (TGF-β), and adhesion molecules (VCAM-1, ICAM-1), thereby promoting microvascular and macrovascular complications. Solid arrows indicate the chronological biochemical steps. Abbreviations: Lp(a), lipoprotein(a); glLp(a), glycated Lp(a); apoB, apolipoprotein B; LDLR, low-density lipoprotein receptor; AGEs, advanced glycation end products; RAGE, receptor for AGEs; ROS, reactive oxygen species; NADPH oxidase, nicotinamide adenine dinucleotide phosphate oxidase; JAK2, Janus kinase 2; STAT, signal transducers and activators of transcription; PI3K, phosphoinositide 3-kinase; AKT, protein kinase B; MAPK, mitogen-activated protein kinase; ERK, extracellular signal-regulated kinase; NF-κB, nuclear factor kappa B; IL-6, interleukin-6; MCP-1, monocyte chemoattractant protein-1; TNF-α, tumor necrosis factor-alpha; TGF-β, transforming growth factor-beta; VCAM-1, vascular cell adhesion molecule-1; ICAM-1, intercellular adhesion molecule-1. Created with https://BioRender.com/tqjwvgv (accessed on 31 October 2025).

Activation of the AGE-RAGE inflammatory axis induces ROS (NADPH oxidase 4) and JAK-2/STAT, PI3K/AKT, MAPK/ERK, and p38 pathways converging on NF-κB, with increased proinflammatory cytokines and chemokines (IL-6, MCP-1, TNF-α, and transforming growth factor-beta [TGF-β]), as well as overexpression of adhesion molecules (VCAM-1, ICAM-1) [130,131,132,133], which induces expression from [134,135,136]. This reinforces the biological plausibility of a glycated Lp(a)-sustained AGE-RAGE cycle in chronic hyperglycemia [128]. See Figure 4.

### 5.5. Atherothrombosis and Antifibrinolysis Measured by Apo(a)

The apo(a) is composed of Kringles, which are also found in prothrombin, factor XII, tissue plasminogen activator (tPA), and urokinase-like activator (uPA) [137]. Plasminogen shares the KV domain, several KIVs, and a protease region with apo(a) [138,139]. Its interaction with fibrin depends on lysine binding sites (LBS) [140,141]. In the KIV-10 of apo(a), an arginine replaces the lysine at position 35, giving it a high affinity for lysine [142]. This change produces molecular mimicry that competes with the physiological fibrin, plasminogen, and tPA complex, generating an abnormal quaternary complex (Lp(a), fibrin, plasminogen, tPA). This alteration hinders the conversion of Glu-plasminogen to Lys-plasminogen and makes fibrinolysis less efficient [143,144,145].

Normally, the ternary complex produces plasmin, which hydrolyzes fibrin and releases C-terminal lysines, amplifying plasminogen activation [146,147]. Apo(a) competes for these residues [148], reducing plasmin formation and clot lysis [149,150].

## 6. Macrovascular Complications

### 6.1. Atherosclerotic Coronary Artery Disease

Atherosclerotic coronary artery disease is a chronic inflammatory process characterized by subendothelial retention of lipoproteins, wall remodeling, and progressive loss of vascular distensibility [151], which culminates in the formation of subendothelial plaques [152]. In DM, the atherogenic dyslipidemia phenotype, along with sustained hyperglycemia, accelerates atherothrombosis by promoting lipoprotein oxidation and glycation [153,154]. In this scenario, Lp(a) is determined by the genetics of the LPA gene, specifically in three chromosomal regions (6q26-27, 9p21, and 1p13) and in two SNPs (rs10455872 and rs3798220) that were strongly related to coronary artery disease [155]. Lp(a) levels ≥ 30 mg/dL (75 nmol/L), and in particular, >50 mg/dL (125 nmol/L), are associated with accelerated progression and an underestimated residual risk of atherosclerotic disease [20].

Clinical evidence supports this link. A prospective meta-analysis reported a higher incidence of coronary events in subjects with elevated Lp(a) [156]. In recent cohorts, elevated Lp(a) was associated with a hazard ratio (HR) of 1.13 (95% CI: 1.04–1.22) for coronary hospitalization or death, after adjustment for LDL-C, reinforcing its contribution to residual risk [153]. In a cross-sectional study in people with T1DM, elevated Lp(a) levels (>120 nmol/L) are associated with a 1.5-fold increased probability of ASCVD, mainly due to coronary events [154]. Taken together, these data support the need to measure Lp(a) to refine risk stratification and prioritize intensive preventive strategies in subjects with DM and/or ASCVD [156].

### 6.2. Ischemic Cerebrovascular Disease

Strokes are one of the most common causes of death and severe long-term disability [157]. Ischemic stroke makes up about 85% of all cerebrovascular events. It happens when blood flow in the brain’s arteries is blocked, which triggers inflammation, excitotoxicity, and damage to nerve cells [158]. In people with DM, factors like endothelial dysfunction, a tendency for blood clots, and lipoprotein oxidation make these inflammatory responses worse and encourage large vessel atherothrombosis [159]. Early detection and prompt treatment are essential for better clinical outcomes [160].

Recent clinical evidence shows that high Lp(a) levels are linked to a greater risk of ischemic stroke [161], in both T1DM [154] and T2DM [160]. Studies using in vivo models have found that Lp(a) can cause endothelial dysfunction and oxidative stress, which worsen vascular inflammation and help cause cerebral infarction [162]. A recent meta-analysis found that people with higher levels of Lp(a) face a greater risk of ischemic stroke [161] and increases gradually according to the number of stenoses [160]. Consistently, a higher concentration of Lp(a) was found in young patients with ischemic stroke, underscoring the importance of this clinical biomarker in ischemic stroke [163]. Additional research indicates that elevated Lp(a) not only increases the likelihood of an initial stroke but also raises the risk of having another stroke and may be linked to poorer recovery outcomes [164]. Overall, this evidence points to Lp(a) as an important factor in both the onset and recurrence of cerebrovascular events, particularly in individuals with DM [160]. Monitoring Lp(a) levels could therefore help doctors more accurately assess risk and tailor strategies to prevent MACE.

## 7. Microvascular Complications

### 7.1. Diabetic Retinopathy

Diabetic retinopathy is one of the leading global causes of visual impairment and blindness, affecting over one million individuals by 2020, with significant regional variation in burden [165]. Clinically, it is defined as a microvascular disease marked by progressive retinal capillary damage that ultimately leads to visual loss [166]. Beyond vascular changes, both retinal inflammation and neurodegeneration contribute to the early stages of the disease [167]. A recent systematic review reported a significant association between elevated Lp(a) levels and progression of diabetic retinopathy [167]. In T1DM with active retinopathy, serum apolipoprotein(a)/Lp(a) levels were higher than in T1DM without lesions and in controls, supporting an association with retinal microvascular complications [168].

Mechanistic pathways include (1) oxidative stress and lipid peroxidation activating the WNT/β-catenin signaling pathway, thereby driving neuropathological changes [169]; (2) vascular tone loss and leakage of modified lipoproteins, contributing to edema and capillary damage [170]; (3) in vivo studies in diabetic rodents showed that injection of highly oxidized and glycated LDL reproduces clinical features of human retinopathy, including vascular leakage, retinal dysfunction on electroretinography, vascular endothelial growth factor overexpression, inflammation, ER stress, and apoptosis [170,171]; and (4) the OxPL fraction of apo(a) has been shown to induce macrophage apoptosis under ER stress, contributing to vascular–neuronal injury and retinal damage [172]. Altogether, these mechanisms support a role for Lp(a) as an emerging mediator of microvascular injury in diabetic retinopathy.

### 7.2. Diabetic Nephropathy

Chronic kidney disease attributable to DM affects 20–40% of patients and represents a leading cause of renal morbidity [173]. Pathophysiological features include mesangial cell proliferation mediated by phospholipase C (PLC), inositol-1,4,5-triphosphate (IP_3_), and elevated intracellular Ca^2+^, which promote glomerulosclerosis [174]. Clinically, the phenotype manifests with microalbuminuria, progressive decline in glomerular filtration rate (GFR), and eventual progression to end-stage renal disease [175]. Lp(a) may modulate these responses, as in vitro studies have demonstrated that elevated Lp(a) levels correlate with mesangial hypercellularity through PLC–IP_3_–Ca^2+^ signaling [176,177]. Concurrently, AGEs binding to RAGEs amplify inflammatory and fibrotic responses, particularly in the presence of glycated LDL, thereby aggravating glomerular injury [178]. Lp(a) metabolism is also altered in diabetic nephropathy, involving reduced renal catabolism [61], increased hepatic synthesis due to protein loss in nephrotic syndrome or peritoneal dialysis [179,180,181], and the impact of inflammation and malnutrition [182]. These processes collectively sustain elevated plasma levels.

Although the causal role of Lp(a) in initiating diabetic nephropathy remains unclear, a meta-analysis reported that elevated Lp(a) is associated with higher odds of nephropathy (OR 1.63) compared with low levels [183]. In people with DM1, higher levels of Lp(a) have also been seen in both early and established stages of kidney disease, suggesting that it could influence kidney progression [82]. Moreover, above the atherogenic threshold of 30 mg/dL, Lp(a) correlates inversely with GFR, with annual declines of 2.75 mL/min versus 1.01 mL/min among patients with low-to-normal Lp(a) [184].

### 7.3. Diabetic Neuropathy

Diabetic peripheral neuropathy affects ~50% of patients and is among the most prevalent diabetic complications [185,186]. Pathophysiology involves Schwann cell metabolic dysfunction induced by hyperglycemia and dyslipidemia, leading to lactate accumulation, oxidative stress, mitochondrial injury, and progressive axonal loss [187]. Elevated Lp(a) levels have been correlated with diabetic neuropathy in T2DM, driven by its proinflammatory, prothrombotic, and oxidative properties [94,186,188]. Chronic hyperglycemia promotes AGE accumulation and endothelial dysfunction, processes that are exacerbated by Lp(a) through inhibition of fibrinolysis, amplification of vascular inflammation, and reduction in blood flow to peripheral nerves [189,190]. Additionally, the lipid-rich composition of Lp(a) contributes to atherosclerosis and ischemia, further impairing neural perfusion [189].

Interestingly, the relationship between Lp(a) and diabetic neuropathy is not consistent across populations. Significant associations have been reported in Asian cohorts, whereas European studies have not confirmed this link [39,186]. These discrepancies likely reflect genetic, metabolic, or environmental modifiers, as well as differences in clinical practice and diagnostic criteria [191]. Further research is needed to clarify the underlying mechanisms and regional variability [186].

## 8. Clinical Utility of Lipoprotein(a) in Patients with Diabetes Mellitus

Measurement of Lp(a) is recommended at least once in a lifetime for the general population to identify individuals with extremely high concentrations (>180 mg/dL or >430 nmol/L) [192]. In ASCVD, the clinically significant threshold is established at >30 mg/dL (75 nmol/L) [33]. In T2DM, Lp(a) has emerged as an independent predictor of cardiovascular risk; elevated levels are associated with up to a two-fold increased risk of recurrent cardiovascular events in patients with established ASCVD [193].

From a clinical standpoint, determination of Lp(a) in diabetic patients enables identification of those at higher residual vascular risk and informs more intensive therapeutic strategies [194]. Nevertheless, the relationship between Lp(a) and diabetes is paradoxical: low Lp(a) concentrations have also been linked to an increased risk of microvascular complications in T2DM, adding complexity to its interpretation [195]. Despite this paradox, current evidence supports Lp(a) as a valuable complementary biomarker that extends beyond traditional cardiovascular risk assessment, with potential implications for personalized risk stratification and the selection of emerging Lp(a)-targeted therapies [33].

Interpretation of Lp(a) should not be performed in isolation but rather in conjunction with traditional risk factors and the patient’s metabolic phenotype [56,196]. In clinical practice, diabetic patients with elevated Lp(a) should be considered for more aggressive lipid-lowering regimens—including statins, ezetimibe, proprotein convertase subtilisin/kexin type 9 (PCSK9) inhibitors, and inclisiran [197,198,199]—and, in the near future, novel agents specifically targeting Lp(a) [200]. In this context, Lp(a) measurement evolves from a static parameter to an integrated determinant of residual cardiovascular risk, capable of guiding therapeutic intensity and informing the implementation of innovative treatment strategies in the diabetic population.

## 9. Therapeutic Perspectives and Future Directions

### 9.1. Conventional Lipid-Lowering Therapies

**Figure 5:**
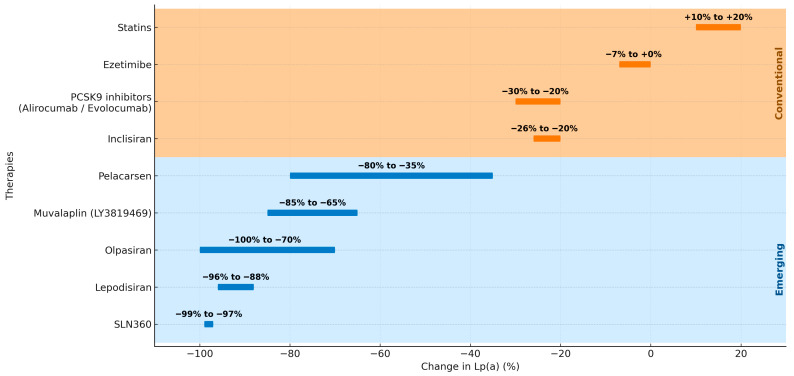
Effect of conventional and emerging lipid-lowering therapies on Lp(a) levels.

Conventional lipid-lowering therapies remain the foundation of dyslipidemia management and cardiovascular prevention [201]. Statins and ezetimibe have historically been the mainstay, achieving 20–55% reductions in LDL-C (as monotherapy or in combination, depending on dose). However, their effect on Lp(a) is null or clinically negligible, limiting their ability to address this residual risk factor [202,203]. PCSK9 inhibitors (alirocumab, evolocumab) and inclisiran have represented a major advance, particularly in patients with DM and ASCVD [197,198,199]. In pivotal trials, PCSK9 inhibitors produced ~50–60% reductions in LDL-C and ~20–30% decreases in Lp(a) [197,198] (Figure 5). Inclisiran demonstrated comparable LDL-C lowering with modest Lp(a) reductions and a twice-yearly dosing regimen that may improve adherence [199]. Taken together, these therapies control LDL-C and partially reduce Lp(a), and therefore constitute first-line treatment for secondary prevention of ASCVD in DM [33]. Recent reviews underscore the need for Lp(a)-targeted strategies [204]. These findings are summarized in Table 1.

### 9.2. Emerging Lp(a)-Targeted Therapies

Therapies that specifically target Lp(a) represent a paradigm shift [33]. Unlike conventional agents, antisense oligonucleotides (pelacarsen) and next-generation small interfering RNAs (siRNAs; olpasiran, lepodisiran, SLN360) have produced sustained Lp(a) reductions of 70–95% [200,206,207,209,210]. In a Phase 2 trial, pelacarsen achieved ~80% Lp(a) lowering in patients with ASCVD [206,207]. The siRNAs (olpasiran, lepodisiran, and SLN360) reported reductions exceeding 94% [200,209,210]. Effects on LDL-C are minimal, with the exception of SLN360, which produced moderate decreases of up to 25% in the Phase 1 APOLLO study [210]. In addition, the oral inhibitor muvalaplin has shown dose-dependent Lp(a) reductions of 45–85% with a favorable safety profile, constituting the first oral approach under investigation for Lp(a) lowering [208,211]. In the context of DM—where elevated Lp(a) is linked to an accelerated progression of complications—these strategies offer a novel and promising avenue. See Figure 5 and Table 1.

## 10. Conclusions

Lipoprotein(a) is a key—yet still underrecognized—determinant of residual vascular risk in diabetes mellitus. Its proatherogenic, proinflammatory, and antifibrinolytic properties link elevated lipoprotein(a) levels to both microvascular and macrovascular complications. Conventional lipid-lowering therapies are insufficient to address this risk, whereas emerging RNA-based agents provide substantial and clinically meaningful lipoprotein(a) reductions. Incorporating lipoprotein(a) measurement and targeted therapies into routine diabetes mellitus care could redefine risk stratification and improve cardiovascular outcomes in this high-risk population.

## Figures and Tables

**Table 1:** Conventional versus emerging therapies targeting Lp(a): impact on lipid profile and cardiovascular risk.

Therapies	Class/Mechanism	Key Trial	Population	LDL-C Reduction %	Representative Reduction % ^ɟ^	Reported Range % ^ɟ^	Timepoint	Key Source (DOI)
Conventional								
Alirocumab (PCSK9-mAb)	Anti-PCSK9 mAb	ODYSSEY (Ph3)	Hypercholesterolemia ± ASCVD	46–61	−25.6	~20–30	24–104 weeks	[197]
Evolocumab (PCSK9-mAb)	Anti-PCSK9 mAb	FOURIER (Ph3)	ASCVD	~59	−26.9	IQR 6.2–46.7	48 weeks	[198]
Inclisiran (PCSK9-siRNA)	siRNA to PCSK9 (semiannual)	ORION-10/11 (Ph3)	ASCVD or equivalent	~50–52	−25.6	~18.6–25.6	Day 510–540	[199]
Statins	HMG-CoA reductase inhibitors	Prospective studies and systematic reviews	Dyslipidemia/ASCVD	20–55	.	n.s. (slightly ↑)	6 weeks–6 months	[202,205]
Ezetimibe	NPC1L1 inhibitors	IMPROVE-IT	Dyslipidemia/ASCVD	18–25 (mono); ~24 add-on	n.s.	~0–7	Weeks–months	[203]
Emerging								
Olpasiran (siRNA-LPA)	siRNA to LPA	OCEAN(a)-DOSE (Ph2)	ASCVD + high Lp(a)	~0–5	−97.4	~70–100	Week 36	[200]
Pelacarsen (ASO-LPA)	Antisense oligonucleotide to LPA	NEJM 2020 (Ph2)	ASCVD + high Lp(a)	~7–26 (2–19 LDL-C-corrected)	−80	~35–80 (dose–dependent)	Weeks 24–26	[206,207]
Muvalaplin (LY3819469)	First-in-class oral small-molecule; Lp(a) assembly inhibitor (apo[a]-apoB100 blocker)	JAMA 2023 (Ph2)	ASCVD + high Lp(a)	n.s.	−75	~65–85 (dose–dependent)	Weeks 4–12	[208]
Lepodisiran (siRNA-LPA)	Long-acting siRNA	JAMA (Ph1)	High Lp(a)	≤5	−94	~88–96	48–50 weeks (to day 360)	[209]
SLN360 (siRNA-LPA)	SiRNA to LPA	APOLLO (Ph1)	High Lp(a)	~20–25 (higher doses)	−98	Up to 98	Day 150–210	[210]

^ɟ^ Reported values of Lp(a). Abbreviations: ASCVD: atherosclerotic cardiovascular disease; LDL-C: low-density lipoprotein cholesterol; Lp(a): lipoprotein(a); mAb: monoclonal antibody; NPC1L1: Niemann–Pick C1-like 1; n.s.: not significant; PCSK9: proprotein convertase subtilisin/kexin type 9; siRNA: small interfering RNA. ↑: slight increase.

## Data Availability

No new data were created or analyzed in this study. Data sharing is not applicable to this article.

## Abbreviations

The following abbreviations are used in this manuscript:

AGEs	Advanced glycation end-products
Apo(a)	Apolipoprotein(a)
Apo(B)	Apolipoprotein B
ApoB100	Apolipoprotein B100
ASCVD	Atherosclerotic cardiovascular disease
ASGP-R	Asialoglycoprotein receptor
CDCA	Chenodeoxycholic acid
DAMPs	Damage-associated molecular patterns
DM	Diabetes mellitus
DR-1	Downregulator of Transcription 1
ER	Endoplasmic reticulum
FGF19	Fibroblast growth factor 19
FXR	Farnesoid X receptor
GFR	Glomerular filtration rate
HbA1C	Glycosylated hemoglobin
HDL-C	High-density lipoprotein cholesterol
HNF4α	Hepatocyte nuclear factor 4 alpha
ICAM-1	Intercellular adhesion molecule-1
IFN-γ	Interferon-γ
IL-1β	Interleukin-1β
IL-18	Interleukin-18
IP_3_	Inositol-1,4,5-triphosphate
IVGTT	Intravenous glucose tolerance test
KIV	Kringle IV
KIV-1	Kringle IV type 1
KIV-2	Kringle IV type 2
KIV-3	Kringle IV type 3
KIV-4	Kringle IV type 4
KIV-5	Kringle IV type 5
KIV-6	Kringle IV type 6
KIV-7	Kringle IV type 7
KIV-8	Kringle IV type 8
KIV-9	Kringle IV type 9
KIV-10	Kringle IV type 10
KV	Kringle V
LDL	Low-density lipoprotein
LDL-R	Low-density lipoprotein receptor
Lp(a)	Lipoprotein(a)
LPA	Apolipoprotein(a) gene
MACE	Major adverse cardiovascular events
MMP-9	Matrix metalloproteinase-9
MODY	Maturity-onset diabetes of the young
NF-κB	Factor nuclear factor kappa B
ox-LDL	Oxidized low-density lipoprotein
OxPL	Oxidized phospholipids
PAF	Platelet-activating factor
PAF-AH	Platelet-activating factor acetylhydrolase
PCSK9	Proprotein convertase subtilisin/kexin type 9
PLC	Phospholipase C
PPARγ/α	Peroxisome proliferator-activated receptors
PRRs	Pattern recognition receptors
ROS	Reactive oxygen species
sdLDL	Dense low-density lipoprotein
siRNA	Small interfering RNA
SNPs	Single-nucleotide polymorphisms
T1DM	Type 1 diabetes mellitus
T2DM	Type 2 diabetes mellitus
TLRs	Toll-like receptors
TNF-α	Tumor necrosis factor-α
tPA	Tissue-type plasminogen activator
VCAM-1	Vascular cell adhesion molecule-1
VLDL	Very-low-density lipoprotein
VLDL-R	Very-low-density lipoprotein receptor
